# Support provided by outreach team leaders to caregivers of HIV/AIDS orphans in the North-West province of South Africa

**DOI:** 10.1186/s12912-024-02282-4

**Published:** 2024-08-31

**Authors:** Boitumelo Joy Molato, Salaminah S. Moloko-Phiri, Magdalena P. Koen, Molekodi J. Matsipane

**Affiliations:** NuMIQ Research Focus Area, School of Nursing, Faculty of Health Sciences, North West University Mahikeng campus, Private Bag X2046 Mmabatho 2745, Mafikeng, South Africa

**Keywords:** Caregivers, HIV/AIDS orphans, Outreach team leaders, Support

## Abstract

**Background:**

The human immunodeficiency virus (HIV) and acquired immunodeficiency deficiency syndrome (AIDS) epidemic have left an overwhelming impact on communities worldwide, particularly in Sub-Saharan Africa, where its effects on family structures are particularly pronounced. Caregivers of HIV/AIDS orphans encounter challenges in fulfilling their caring duties. Consequently, they rely on the outreach team leaders (OTLs) for support to care for HIV/AIDS orphans.

**Aim:**

This study aimed to explore and describe support provided by OTLs to caregivers of HIV/AIDS orphans in the North West Province of South Africa.

**Methods:**

The exploratory, descriptive, and contextual design meaning the study was conducted was in the contexts where caregivers of HIV/AIDS orphans reside. The study was conducted in five local municipalities in the Ngaka Modiri Molema district of the North West Province of South Africa. Ward-based outreach nurses were participants in the study. Semi-structured focus group interviews were used for data collection. Thematic analysis was used to analyze data. Throughout the study, ethical principles were adhered to. The study also adhered to four trustworthiness principles: credibility, confirmability, transferability, and dependability.

**Results:**

Three main themes emerged from this study: the conduction of home visits to caregivers of HIV/AIDS orphans, the coordination of a multidisciplinary team for support, and the facilitation of support groups.

**Conclusions:**

This study revealed that that caregivers of children orphaned by HIV/AIDS benefitted from the support provided by OTLs in the North West province of South Africa. The support provided by OTLs harnessed positive relationship between caregivers and children orphaned by HIV/AIDS.

**Supplementary Information:**

The online version contains supplementary material available at 10.1186/s12912-024-02282-4.

## Introduction

The Human immunodeficiency virus (HIV) continues to be a serious global public health concern, accounting for 40.4 million [32.9–51.3 million] deaths worldwide [1]. At the end of 2022, there were an estimated 39.0 million [33.1–45.7 million] people living with HIV (PLWHIV), with 25.6 million of those individuals living in the World Health Organization’s (WHO) African Region [1]. There were 1.3 million [1.0–1.7 million] new HIV infections and 630 000 [480 000–880 000] deaths from HIV-related causes in 2022 [1] Globally, this pandemic has claimed countless lives of men and women and left many children as orphans. An estimated 143 to 210 million children are orphaned worldwide; that number grows by about 5,760 children every day [2]. In 2022, Mozambique was the only country in the Southern African Development Community (SADC) to record 1.30 million of children orphaned by HIV. The second highest country in SADC to record high number of orphaned children by HIV was Tanzania with 959, 030.00. The Republic of South Africa (RSA) as the third highest country recorded 717, 017.00 [3]. These orphans are left by parents without resources [4]. Subsquently, it will be the responsibility of the primary caregivers to see to it that these orphans are well taken care of. The term “primary caregivers” describes individuals who are in charge of giving care to someone who is unable to take care of themselves [5]. Under normal circumstances, primary caregivers may be brothers, sisters, aunt, uncles, extended families and sometimes members of the community. Previous studies revealed that caregivers of HIV/AIDS orphans face challenges that include a lack of support from social services and family, stigma, and discrimination when caring for HIV/AIDS orphans [6, 7].

Due to the high rate of unemployment, caregivers are unable to provide orphaned children with basic needs such as access to necessities like food and clothing [8, 9].To help caregivers with some of the financial challenges involved in raising orphans, the South African government offers social security in the form of Foster Care or Child Support. Notwithstanding the restricted financial resources, poverty continues to be a significant issue that impacts orphaned children both before and after a parent passes away [8]. With regards to the older caregivers, caregiving is not easy due to age they face many difficulties that include the execution of duties without undergoing proper training for caregiving.

The authors further claimed that the challenges that older caregivers face predispose them to burdens that affect their physical, and psychological well-being [10, 11]. Furthermore, grandparent caregivers often present with health challenges, such as poorer emotional well-being and declining psychological health, because of stressors arising from caring for their grandchildren [12, 13]. Consequently, the stress of providing care exposes caregivers to long-term illnesses that make it difficult for them to perform their daily caregiving responsibilities [14]. Given these challenges, the South African (S.A.) government introduced primary health care (PHC) re-engineering strategy to promote health by providing services to society at their respective homes [15].

The PHC re-engineering model comprises ward-based primary health care outreach teams (WBPHCOT), district specialist teams, and school health teams [16]. The author further indicates that this strategy represents an attempt to formalize, standardize, and integrate community-based services into the PHC system. The WBPHCOTs act as a link between healthcare facilities and the communities they serve [17, 18]. This can be particularly helpful in rural places where people must travel great distances for medical care and when communities are underserved and disadvantaged. The purpose of PHC re-engineering is to establish a district health system focused on solving society’s most pressing health needs through PHC teams [19]. Each ward has a WBPHCOT led by a professional nurse commonly known as an outreach team leader (OTL).

In the South African setting, the OTLs are considered community health nurses because they provide health education, encourage behavioural changes that empower people to take charge of their health, and promote health within the community [20–22]. The activities performed by OTLs include household and individual assessments, identification of problems, provision of interventions, and referrals [19]. These routine visits enable OTLs to conduct different assessments of the individuals they visit. The OTLs conduct assessments on caregivers of HIV/AIDS orphans; they intervene and provide the needed support.

The OTLs as the leaders of the WBPHCOT work closely with community health workers (CHWs) who are non-healthcare professionals. In the United States, CHWs play an essential role in the treatment of diseases like hypertension, diabetes, HIV, cancer screening, and lowering the risk of cardiovascular disea [23–26] In South Africa, CHWs provide the support that contributes to the successful implementation of the human immunodeficiency virus (HIV) program [17, 27]. Up to now, the support provided by OTLs in the North West Province (NWP) of South Africa (SA) remains unknown regardless of the challenges faced as reported by existing literature [28]. Majority of the studies on WBPHCOT were conducted in the South African context. These studies were mainly focusing on challenges and future directions, its effect on PHC performance, barriers to its implementation, and the promise and and reality of conducting Tuberculosis (TB) household contact tracing [29–32].

Regardless of the existence WBPHCOT caregivers continue to render care to HIV/AIDS orphans under stressful circumstances that lacks support from all corners of life. Individuals’ sense of belonging and overall health and well-being are greatly enhanced by support. Support can be provided in different forms, including physical, emotional, psychological and even spiritual. People who receive support are able to handle life’s obstacles and stress. Therefore, the researchers found it necessary to explore and describe the support provided by outreach team leaders in the North West province of South Africa. This study addresses the third objective of the main (PhD) study which aims to develop health promotion strategies to improve the health and well-being of caregivers of HIV/AIDS orphans in NWP of SA. Exploration of the support provided by OTLs may assist in formulating recommendations that focus on mitigating circumstances surrounding caregivers of HIV/AIDS orphans. Furthermore, the findings of this study might assist in developing health promotion strategies as the main objective of the study.

## Materials and methods

### Research design

This study used the qualitative exploratory, descriptive design to explore and describe the support provided by OTLs to the caregivers of HIV/AIDS orphans in the NWP of SA. Through qualitative research, researchers were able to get insight into the meaning that people attribute to their experiences by getting access to research participants’ thoughts and feelings [33]. Exploratory research design was used to better understand the nature of the problem because little was known regarding the phenomenon [34, 35]. Descriptive research aims to shed light on current issues or problems through a data collection process that allows them to be fully descriptive and go into detail about the phenomena [36]. Moreover, the descriptive research design was used to capture the most authentic and accurate description of data collected to describe the support provided by OTLs to the caregivers of HIV/AIDS orphans in Ngaka Modiri Molema district in the NWP of SA. concerning contextual research design, we interacted with the caregivers in the village in which they reside [37]. Furthermore, the environment where data was collected was free from manipulation natural surroundings entails that the environment is free from manipulation [38].

### Study setting

The study was conducted in five local municipalities of the Ngaka Modiri Molema District of the NWP of SA. The local municipalities where the study was conducted were Mahikeng, Ratlou, Ramotshere Moiloa, Ditsobotla, and Tswaing. The district shares an international border with Botswana and is located at the center of the province.

### Population and sampling

The population of the study was OTLs rendering care to the caregivers of HIV/AIDS orphans in Ngaka Modiri Molema District. The OTLs considered eligible for this study were registered nurses with a qualification in community nursing, with or without additional qualification of PHC, and had the experience of at least six months working as OTLs. Non-probability purposive sampling was used to sample the OTLs who were eligible to actively participate in the study. Every participant in the target population does not have equal chances because the sample population was chosen in a non-systematic manner [39]. Purposive sampling assisted in selecting participants who shared fundamental information related to the study [40].

### Recruitment of the participants

Approval letter from North West University Health Research Ethics (NWUHREC) was submitted to the North-west Provincial Department of Health to seek permission to conduct the study in five local municipalities of Ngaka Modiri Molema district. After obtaining permission, pamhplets were posted on the notice boards to recruit OTLs to participate in the study. The pamphlets contained the contact details of the researcher, promoters, and the research assistant. All participants who participated in this study called the research assistant for more information regarding the study this include making appointments to sign informed consent and data collection. Recruitment was done by the research assistant.

### Data collection

Data was collected face-to-face at the local clinics in five local municipalities of the Ngaka Modiri Molema district of the NWP. Five (5) focus group sessions were conducted to achieve the objectives of the study. The OTLs were asked to choose suitable place for data collection and they could not find it based on that the researcher proposed a neutral healthcare facility that is accessible for all. Three focus groups in three municipalities were having five (5) participants whilst two focus groups from two different municipalities had six (6) participants each. The OTLs who were using vehicles to come to data collection and they produced invoices of the petrol/diesel purchased so that they can be reimbursed. Interviews were conducted in five clinics of all municipalities where data was collected. The study used semi-structured focus group discussions to gather information from the OTLs. The interview guide was developed to achieve the objectives of the study. The exploratory question was: What support is provided by OTLs to caregivers of HIV/AIDS orphans in NWP, SA? An audio recorder was used to record the discussions. The discussions lasted for 45–60 min. To accomplish the goals of the study, open-ended and brief questions were asked [41]. Field notes were used to capture all activities that unfolded during the discussions to ensure accurate recording of vital information [42]. Interviews were conducted in English and data saturation was reached in focus group number five.

### Data analysis

The researcher and independent co-coder used six (6) steps of thematic analysis to analyze data as stipulated by [43]. In terms of familiarisation of data, which is the first step, we have read and re-read the transcripts to familiarize ourselves with the data. The second step of thematic analysis followed was the generation of initial codes [43, 44]. The data was arranged in a methodical and comprehensible manner. After that, we each started coding a transcript on our own. After we were done, we reviewed, compared, and adjusted our codes before going on to the remaining transcripts. The quotations that emerged from the interviews were used to cite the findings.

In the third step which is search for themes, when we looked at the codes, it was evident that some of them fit into a theme. The codes were arranged into more general themes after this process, which appeared to address a particular aspect of the research question [43, 44]. Concerning the fourth step which is reviewing themes, this stage involves going over, revising, and expanding on the initial topics that we determined in Step 3 to check coherence.

After reading the information related to each theme, we evaluated if the information supported the theme. In terms of the fifth step which was to define themes, we determined the fundamental idea of the theme as well as its main points. This was followed by evaluating each sub-theme in terms of its relevance to the main theme and its interactions with other sub-themes [43, 44]. Writing up was the sixth and the last step, before writing the report, the researcher and co-coder met to check if they had similar themes and sub-themes of the study.

### Trustworthiness

The researcher ensured trustworthiness by adhering to the four principles of trustworthiness namely, credibility, transferability, confirmability, and dependability [45].

#### Credibility

The researchers interacted with the participants for a long time to know their viewpoints. The rationale behind interaction was to allow them to guarantee the accurate representation of their perspectives and experiences. Reflexivity was upheld throughout the data collection, analysis, and interpretation stages to reduce bias in the findings. Numerous methods of gathering data such as interviews and field notes were employed to verify information from multiple perspectives, strengthening the veracity of the descriptions [45].

#### Transferability

A thick description was maintained by fully outlining the research setting, participants, and techniques. To ascertain whether the results would be relevant or transferable to comparable populations or circumstances outside the study context, the sampling techniques that were employed were explained, along with the reasons for participant selection [45].

#### Confirmability

The participants were checked to review and confirm the correctness of data to improve confirmability. Biasedness was reduced by discussing interpretations and findings with co-workers or experts [45].

### Ethical considerations

This study was approved by Quality in Nursing and Midwifery (NuMIQ), a research focus in the Faculty of Health Sciences at the North West University (NWU). Ethical clearance was obtained from the NWU Health Research Ethics Committee (NWU-00196-21-A1). The North West Department of Health gave permission to conduct the study. The informed consent was obtained by the research assistant. After the participants show interest to participate in the study, the researcher forwarded them the consent form through email, and whatss app platform. Participants who did not have access to any platform to receive the consent form, were approached physically. Moreover, the participants were given at least 7–14 days to make an informed decision before signing the consent form. After 7–14 days, the researcher and the research assistant met with the participants face to face to explain further and for signing of the consent form.

Before conducting the study, participants were given a full explanation regarding the purpose of the study and how the entire process would unfold. The participants were told about their right to participate voluntarily in the study without being coerced. Furthermore, they were also told about the right to terminate participation anytime when they wishes and that they should not expect any form of reward for being part of the study. The principle of privacy was maintained by placing ‘‘Do not disturb session in progress’’ at the door, and anonymity principle was maintained by not using the participants’ names. The collected data was shared only with the research team.

## Results

A total of 27 professional nurses from five local municipalities voluntarily participated in this study. Both genders were represented although the majority of the participants were females see Table 1 for more details.

**Table 1:** Demographic information of outreach team leaders

Variable	Category	Number
Gender	Male	4
	Female	23
Age in years	30–39	02
	40–49	05
	50–59	12
	60–69	08
Nurses participating in the study per local municipality	A B C D E	06 05 05 05 06
	**Total**	**27**

A total of five semi-structured focus group discussions consisting of five to seven participants were conducted face-to-face see Table 2 for more details regarding the findings of the study. Three main and ten (10) subthemes were identified in the study.

**Table 2:** Findings of the study

Main themes	Sub-themes
1. Conduction of home visits to the caregivers of HIV/AIDS orphans.	1.1 Promotion of medication adherence among HIV/AIDS orphans. 1.2 Adoption of a Child Strategy. 1.3 Performance of routine blood and growth monitoring of HIV/AIDS orphans. 1.4 Provision of physical support to the caregivers of HIV/AIDS orphans during disclosure.
2. Coordination of multidisciplinary team support.	2.1 Referral to psychologists 2.2 Referral to social workers 2.3 Referral to the Dieticians/Nutritionist for nutritional support. 2.4 Referral to the law enforcement officers for security support.
3. Facilitation of support groups for both caregivers and children orphaned by HIV/AIDS.	3.1 Adherence clubs. 3.2 Provision of adolescent-friendly services.

Three (3) main themes and ten (10) subthemes were identified in the study. To the researchers’ knowledge, this study appeared to be a new contribution to the body of knowledge. The WBPHCOT leaders highlighted different types of support that they provide to caregivers of HIV/AIDS orphans. The support provided by OTLs included conducting home visits to the caregivers of HIV/AIDS orphans, coordinating multidisciplinary teams for support, and facilitating support groups for both caregivers and HIV/AIDS orphans. Each support provided by outreach team leaders was broken into subthemes and is discussed below.

## Results

### Theme 1.1 conduct home visits at homes of the caregivers of HIV/AIDS orphans

The first theme identified was conducting home visits at the homes of caregivers of HIV/AIDS orphans. Four (4) sub-themes emerged from this theme, namely, promotion of medication adherence among HIV/AIDS orphans, adoption of a child strategy, performance of routine blood and growth monitoring of HIV/AIDS orphans, and provision of physical support to the caregivers of HIV/AIDS orphans.

### Sub-theme 1.1 promotion of medication adherence among HIV/AIDS orphans

The promotion of medication adherence emerged as the first sub-theme. Participants used viral load suppression as a tool to determine whether caregivers give HIV/AIDS orphans medication accordingly. For example, if they encounter such cases, they visit them at their homes to conduct assessments to exclude the underlying causes of the viral load. During home visits, the outreach team leaders conduct their assessment to confirm if the caregiver gives the HIV/AIDS orphan medication accordingly. This was expressed as follows:

*One of our primary role and responsibility as OTLs is to assess medication adherence among HIV/AIDS orphans during home visits. If there is medication non-adherence*,* it will be an indication of viral load monitoring for viral suppression.* (FG3 Participant C)

One of the participants reported that they used health education as a weapon to prevent viral suppression and said:

*The OTLs put more emphasis on health education to avoid reaching the point of poor viral suppression. If there is no viral suppression*,* CHWs are incorporated to initiate HIV/AIDS orphans on the DOT strategy.* (FG 3 Participant A)

CHWs are involved in the cases of medication non-adherence to initiate the DOT strategy. The following was echoed.

*If there is no improvement in medication adherence*,* the outreach team leaders as champions of the programme request the CHWs to take over from caregivers by implementing the DOT strategy. The CHWs will be expected to visit the caregivers’ homes every time to ensure that the child takes treatment as s/he should. This strategy helped us a lot to improve adherence among orphaned children. Also this strategy equipped caregivers with more skills because the CHWs involved them during the DOT process.* (FG 1Participant D).

### Sub-theme 1.1.2: Adopt a child strategy

Adopt a child strategy was identified as a second subtheme in which outreach team leaders CHWs allowed participants to adopt one HIV/AIDS orphans to monitor whether the caregiver gave the child medication accordingly and to ensure that other aspects of caring were adhered to. The participants expressed the following:

*There is another strategy introduced by District Health called the Adopt-a-child strategy. About this strategy*,* the OTLs and CHWs are expected to adopt a child each. Both OTLs and CHWs follow them up at home*,* and this strategy motivates the caregivers to better take care of the HIV/AIDS orphans. During home visits*,* we check if the caregiver gives the orphan the correct medication*,* dose*,* time*,* and route.* (FG 1 Participant C).

Another participant added,

*The adopt-a-child strategy is part of physical support because we assess if the child is growing well*,* gaining weight*,* and improving on physical appearance. Adherence has improved; …before the implementation of the Adopt a Child strategy*,* caregivers did not know how to give medication*,* and they did not report to us. Currently*,* caregivers are doing the right thing*,* and we are following up regularly to ensure they don’t deviate.’* (FG 2 Participant B).

Another participant outlined the significance of the strategy. She said:

*In general*,* this adopt-a-child strategy capacitates the caregivers of HIV/AIDS orphans with skills because after we adopt a child*,* we start taking care of them; we even do follow-ups at their respective places to see if they are well and getting treatment correctly. After some time*,* we handed the child over to CHWs for follow-ups at home to see if the caregivers were doing precisely what we did. The feedback we receive from CHWs is positive that caregivers are doing well in giving medication*,* there is a positive relationship between orphans and caregivers*,* and caregiving skills are enhanced. (*FG 3 Participant E).

### Sub-theme 1.1.3: performance of routine blood and growth monitoring of HIV/AIDS orphans

Monitoring of growth and routine blood checks was identified as a third sub-theme. The outreach team leaders are expected to perform routine blood tests at the respective homes of the caregivers of HIV/AIDS orphans. The following was expressed by one of the participants:

*After adopting a child*,* the CHWs will be expected to borrow a weighing scale at the nearest clinic to weigh the child at home. As OTLs*,* we take routine blood checks at home and attach the child to mentors who are CHWs to ensure that the child regularly gets food and do pill counts to measure adherence.* (FG 3 Participant C).

Another participant expressed the following:

*If the child does not show any progress*,* we visit the family every day for at least three months to identify the root cause of the problem. After that*,* we take routine blood checks to see if there is viral suppression.* (FG 1 Participant A).

Another participant added:

*We are closely working with CHWs. They have been capacitated with knowledge and skills related to caring. If we come across underweight children during our home visits*,* we refer them to CHWs to analyze their immunization cards to see whether the child is not malnourished.* (FG 2 Participant D).

### Sub-theme 1.1.4 provision of support to caregivers of HIV/AIDS orphans during disclosure

Supporting caregivers of HIV/AIDS orphans during disclosure was identified as the fourth subtheme. The participants reported that it was the responsibility of the caregivers to disclose their status to HIV/AIDS orphans. As participants, they avail themselves of support when caregivers disclose their status to HIV/AIDS orphans. The following was verbalized:

*Nurses do not disclose HIV status*,* and caregivers are the ones who should do that. As nurses*,* we avail ourselves just for support. The orphan was already a teenager because she was 15 years old. Luckily*,* we managed to engage the grandmother because our concern was that the orphan was not receiving treatment. We advised the grandmother to disclose her status to her and to motivate her to start treatment by taking her chronic treatment in her presence so that the child can see that she is not the only one taking treatment alone.* (FG 2 Participant B).

Another participant shared the sentiment of providing support during disclosure. She said:

*We avail ourselves for support when they disclose HIV status to the orphans. The orphans should not hear about it from people in the village they are not related to. If the child can hear about that from other people*,* the matter will not sit well with them*,* and that will cause them to be angry at the person who was supposed to have disclosed the status.* (FG 1 Participant B).

Another participant verbalized:

*If I educate the caregivers of HIV/AIDS orphans about the disease of the child*,* it is her responsibility to disclose HIV status to the child in my presence. In principle*,* it is not the responsibility of nurses to disclose their HIV status to the children. The people who are allowed to disclose their HIV status to children should either be caregivers or parents.* (FG 3 Participant B)

#### Theme 2.1 coordination of multidisciplinary team for support

Five (5) sub-themes emerged from this theme. These include liaison with counselors for HIV testing, counseling, and disclosure, referral to the social worker for support, referral to the dieticians/nutritionist for nutritional support, and referral to the law enforcement officers for security support.

### Sub-theme 1.2.1 referral to psychologists

The challenges faced by caregivers of HIV/AIDS orphans affect their strengths in executing caring responsibilities. The participants reported that if they come across such cases, they refer the caregivers of HIV/AIDS orphans to psychologists. One of the participants echoed the following:

*All these overwhelming circumstances faced by caregivers affect their psychological well-being e.g.*,* if you do not have food at home*,* and on the other hand you must give children treatment this automatically affects the psychological well-being therefore we will be forced to bring the service of psychologist for counselling.* (FG 1 Participant A).

The same sentiment was shared by one of the participants who said:

*In the cases whereby caregivers are unable to cope*,* we rope in the psychologist because some of these people need intensified counselling from the specialist who can take them through the whole process of counselling. I don’t think social work and psychologist duties are the same*,* but I would say psychologist can intervene better in this matter.* (FG 3 Participant A).

The participants also refer caregivers of HIV/AIDS orphans who are not willing to disclose their HIV status to the children. The following was echoed:

*To add to what my colleague has just presented*,* disclosure is done by caregivers or parents but if she is reluctant to disclose*,* they involve us. There are psychologists in clinics. They do have schedules for visiting clinics. We refer both caregiver and a child to the psychologist to empower him/her with relevant knowledge and skills on how to disclose the status to the child.* (FG 2 Participant E).

### Sub-theme 1.2.2 referral to the social workers for support

Referral to the social workers for support emerged as the second subtheme. Participants reported that they worked closely with social workers to refer cases where caregivers of HIV/AIDS orphans were not registered for social grants and when the grants were not utilized appropriately. One of the participants expressed herself as follows:

*Some caregivers are misusing the grants. You will find that when we conduct home visits*,* children are not well taken care of*,* and the child has lost weight and is wearing old clothes just because the caregivers buy or use the grants to buy liquor. If we come across such cases*,* we involve social workers to intervene so that the grants can be given to the relevant people who are taking care of the HIV/AIDS orphan full time.* (FG Participant 2 C).

Another participant echoed the following:

*I think I have mentioned the social worker role where we*,* as nurses*,* cannot win the challenging situation; we seek their input and services. If some do not have grants*,* some don’t have birth certificates and road to health charts*,* and these are the documents needed to apply for grants. When all these documents are gathered*,* it will be easy for the social worker to recommend them for grants.* (FG 1Participant D).

One of the participants supported the sentiment as follows:

*Usually*,* we compile a list for maybe three months to provide food parcels to*,* for example*,* Family A*,* B*,* and C*,* and in the next cycle*,* we give to Family D*,* E*,* and F. Social workers have profiles of all families. In this case*,* they will be able to know whether families that are provided with food parcels deserve that privilege.* (FG 3 Participant C).

### Subtheme 1.2.3 referral to the dieticians/nutritionist for nutritional support

Referral to the Dieticians/Nutritionist for nutritional support was identified as the fourth subtheme. Participants reported that caregivers unable to buy food are referred to dieticians for food parcels. One of the participants expressed the following:

*The dieticians came in when we assessed the families and found out they were not eating well. We then invite the dietician to come and conduct further assessment and recommend food parcels if need be. In cases where a child is not growing well*,* the child will be placed on a special diet for maybe a month to see if s/he cannot pick up weight.* (FG 1 Participant E).

Orphaned children who are malnourished are also referred for supplements. The participants echoed the following:

*We also have a team of nutritionists and dieticians assisting us here in the sub-district. When we have a child of poor nutritional status since caregivers experience many challenges*,* we then involve them to intervene. They assess them first and offer some food parcels to help improve the child’s weight.* (FG 3 Participant A).

Another participant added:

*Regarding HIV/AIDS orphans who are not thriving well*,* we always involve nutritionists as members of a multidisciplinary health team to intervene by putting them on the correct diet and food parcels that will improve body weight so that it can be interpreted in line with age.* (FG 2 Participant C).

### Subtheme 1.2.4 referral to the law enforcement officers for security support

Referral to law enforcement officers for security support emerged as a subtheme where participants reported that when they come across cases whereby HIV/AIDS orphans are being abused, they refer such cases to the police for further investigation and management. The following was echoed:

*If the orphan is abused*,* it is the prerogative of the social workers to involve the South African Police Services to establish the root cause of abuse and further management.* (FG 2 Participant F).

One of the participants stated that they also involve the police when the needs of the children are neglected and said:

*One of the common cases is child neglect. Orphans are cared for by relatives when their parents pass away. So*,* if their stay with relatives is unstable*,* for example*,* they move from one family to another for two months with a cousin and two months with an uncle and aunt. It is challenging to decide who is entitled to a foster care grant. For example*,* the uncle took the foster care grant during the child’s stay at his home and used the funds to buy his own needs and ignored to buy clothes and other essentials for the orphan. Therefore*,* we involved the police and social workers to intervene.* (FG 3 Participant D).

One of the participants emphasized the importance of law enforcement and said:

*There are recreational places adolescents and adults like visiting. In those places*,* some of them are killed*,* some raped*,* and some stay there for quite some time without coming back home. All these problems require people who can do follow-up*,* and in this case*,* police officers are the most relevant agents to do that.* (FG 3 Participant A).

Another participant reported:

*You will find that some caregivers do not report cases of abuse of HIV/AIDS orphans. When we encounter such cases*,* we do a thorough assessment before involving social workers. She will have to confirm her assessment as well. After assessments*,* there will be a need to involve law enforcement officers.* (FG 3 Participant D).

#### Theme 1.3 facilitation of support groups for both caregivers and HIV/AIDS orphans

Two (2) subthemes emerged from this theme, including adherence clubs and adolescent youth-friendly services.

### Sub-theme 1.3.1 adherence clubs

Adherence clubs were identified as the first subtheme in this study. The adherence clubs are reserved for all patients who are on chronic treatment. The initiative aimed to reduce overcrowding at the facilities by scheduling the exact dates for treatment collection for both parties. Adherence clubs assisted caregivers in readily disclosing their status to orphans without postponing. One of the participants indicated:

*The Department of Health has introduced a project called Adherence Clubs. In the cases of both caregivers and the orphans taking treatment*,* for example*,* chronic treatment and ART*,* we schedule the same follow-up dates for both. The caregivers benefit from this project of adherence clubs because they do not struggle to disclose their HIV status to the orphans. If the HIV/AIDS orphan asks questions*,* the caregiver just responds shortly by saying*,* ‘I am also taking treatment’. Most of the time*,* we encourage caregivers to take their medication at the same time as the orphans. There has been huge progress regarding disclosure since this initiative was implemented.* (FG 3 Participant B).

One of the participants elaborated more on the terms of reference of adherence clubs. They also address the needs of patients who are on other treatments. She said:

*We have initiated adherence clubs that are yielding positive results in the villages. These adherence clubs are not only for people who are HIV positive. They are for all chronic patients. They end up seeing each other at the clubs. We pack treatment according to a patient’s needs so no one can judge another regarding their treatment. As a result*,* it makes things easier for us. We don’t write about the disease that one suffers from nor the treatment that individual takes. During visits to adherence clubs*,* we give health education on specific topics. It is effective and impacts caregivers*,* and they become free from stigma and discrimination.* (FG 1 Participant C).

The club has played a fundamental role in improving adherence to treatment and the attitude of the community towards caregivers of HIV/AIDS orphans. The following was echoed:

*The club has improved adherence to treatment because it facilitates the process of patients accepting themselves. As caregivers of orphaned children*,* adherence clubs have helped to reduce the stigma and discrimination. The club has made all categories of people collecting treatment to relax after noticing that they are not alone in taking chronic treatment.* (FG 2 Participant F).

### Subtheme 1.3.2 adolescent youth friendly services (AYFS)

The AYFS emerged as the subtheme in this study. The participants indicated they started a support group for adolescents at the facilities. The support group also discusses activities such as health education and other issues affecting adolescents’ health. One of the participants mentioned:

*Yeah*,* the other point we did not mention is that we also have teenager clubs. We invite them*,* and they can come along with caregivers to the clinic. We set a date and time*,* and when they arrive*,* we carry out activities with them*,* including how to get treatment and the importance of adhering to treatment. The adolescents are bound to come along with caregivers*,* and I think the initiative is productive.* (FG 1 Participant D).

The main aim of initiating adolescents’ clubs was to create a platform where matters that are related to HIV and affect the health of adolescents are ventilated. One of the participants echoed the following:

*The main purpose of this teen club is to make sure that these teenagers accept their status and that they can teach those who are not HIV positive about the disease and to accept the status if the outcomes of HIV counselling and testing are positive. Again*,* teenagers’ understanding of the disease makes the lives of caregivers very easy because caregivers sometimes struggle to convince teenagers to accept their status; therefore*,* teen clubs play a major role in motivating both teenagers and caregivers. There will be a good working relationship at home*,* and confidentiality will not become an issue when the child is affiliated with teen clubs.* (FG 1 Participant C).

The teenagers are also advised to attend with caregivers of HIV/AIDS orphans, and the sessions that are conducted regularly boost their confidence. One of the participants echoed the following:

*At the AYFS*,* we capacitate adolescents with knowledge and skills regarding different conditions like HIV*,* STIs*,* and other chronic conditions. The sessions empower them with knowledge that guides them on how to conduct themselves when they have tested HIV positive. It also assists caregivers in disclosing their HIV status to children on time. If there is a delay in disclosure*,* children will start asking the caregiver many questions like ‘What is the medication that I am taking? Why am I taking the medication?’ This will embarrass the caregivers*,* but this will ultimately compel the caregivers to disclose their HIV status to the child.* (FG 3 Participant E).

## Discussion

This study aimed to explore and describe the support provided by OTLs to the caregivers of HIV/AIDS orphans. To our knowledge, this study is a new contribution to the body of knowledge. The objective of this study was successfully met, and three (3) main themes emerged from this study, namely the conduction of home visits to the caregivers of HIV/AIDS orphans, the coordination of multidisciplinary teams for support, and the facilitation of support groups for both caregivers and HIV/AIDS orphans.

This study revealed various support that is provided by OTLs to caregivers of HIV/AIDS orphans. Conducting home visits at the homes of the caregivers of HIV/AIDS orphans emerged as the primary theme, and subthemes were also identified in this study. Many of the OTLs reported that conducting home visits assisted them in identifying orphaned children not adhering well to medication. In such cases, OTLs assigned CHWs to implement DOT on caregivers who are not giving orphaned children medication accordingly. Researchers defined DOT as a strategy that non-healthcare professionals use to observe whether the patients take treatment [46].

The findings of this study are consistent with the outcomes of the study conducted in Zimbabwe that reported that nurses involved CHWs when there were instances of ART non-adherence among HIV/AIDS orphans [42]. The authors went on to establish that during home visits, CHWs work closely with caregivers to identify any barriers that might prevent adherence to medication. Following this results from a systematic review and meta-analysis also confirmed that DOT was beneficial to people who were more non-adherent to ART [47]. The authors also suggested that when employing DOT on people who exhibit non-adherence behavior, steps should be taken to ensure adherence.

These steps of ensuring adherence to ART were evident in the study conducted in Zimbabwe, CHWs visited the caregivers of HIV/AIDS orphans at home to follow up on matters that arose from scheduled clinical appointments and provide them with adherence support [48]. Existing literature and the current study categorically described the importance of the DOT strategy. Within the context of this study, the strategy improved adherence among HIV/AIDS orphans and capacitated caregivers with skills on how to maintain and sustain adherence.

Each one of the OTLs was obliged to adopt one HIV/AIDS orphan from each family and employ a holistic approach to ensure that the needs of that orphan are well taken care of. In line with the literature, the study conducted in SA revealed that CHWs used a community-based intervention strategy to supervise caregivers of HIV/AIDS orphans on medication giving by visiting them weekly in their respective households [49]. Similar findings were reported in Uganda, where the Grandparents Action Support Project (GAS) was established to enhance the capacity of older caregivers to provide care to HIV/AIDS orphans and to improve the overall welfare of the families taking care of these children [50]. This study’s evidence demonstrated the strategy’s broad benefits, including the development of caring abilities for caregivers of HIV/AIDS orphans. The adopt-a-child approach has made it easier for OTLs, CHWs, caregivers, and HIV/AIDS orphans to have healthy relationships.

Routine blood and growth monitoring played a fundamental role in assessing viral suppression among HIV/AIDS orphans. In concurrence, the cross-sectional study conducted in Tanzania reported that the households of caregivers of HIV/AIDS orphans that were monitored by Community case workers (CCWs) were more likely to be virally suppressed than those who did not [51]. The authors continued by asserting that there was a 149-fold increase in the likelihood of viral suppression in HIV/AIDS orphans who adhered to ART at a rate of > 95% compared to those who did not. In this study, the OTLs visited HIV/AIDS orphans daily who did not thrive well or were malnourished.

During home visits, the OTLs assess how caregivers give medication and other aspects of a healthy lifestyle. After three (3) months, OTLs take routine blood monitoring to assess if the viral load is being suppressed and if the CD4 count is going up, as both are elements of growth monitoring and medication adherence. The literature revealed that orphaned children with viral suppression always present with improvement in clinical well-being, weight gain, and reduction of opportunistic infections [52]. Researchers whose focus was on low- and middle-income countries viewed viral load monitoring as a useful tool for identifying children living with HIV who require extra adherence assistance, minimizing regimen switching, and maintaining treatment alternatives [53]. Based on the findings of this study and existing literature, we assume that optimal adherence to ART play is of paramount importance to improve CD4 count and suppress the viral load of HIV/AIDS orphans. It is evident in this study that OTLs had measures in place to support caregivers to sustain adherence.

Another interesting finding revealed by this study was that the OTLs were responsible for providing caregivers of HIV/AIDS orphans with any kind of support during disclosure. In line with previous research, a qualitative study carried out in the Eastern Cape of South Africa found that healthcare workers (HCWs) used a variety of techniques to assist caregivers with disclosure [54]. These included talking with caregivers to find out what their primary disclosure challenges were and teaching them how to disclose and handle the emotional responses of children after disclosure [54]. The cross-sectional survey about health care workers (HCWs) perspectives on disclosure to HIV-infected children conducted in Gauteng and Mpumalanga Provinces of South Africa revealed different results regarding who is entitled to disclose [55]. According to the survey, 48,5% of respondents felt that caregivers should take the initiative to disclose, 42.7% believed that caregivers and HCWs should share responsibility for disclosure, and 8.8% said that HCWs should take the lead in disclosure [55].

Up to now, it is not clear about who should conduct disclosure between HCWs, and caregivers based on existing literature. Contrary to the results of the current study, a qualitative study carried out in Zimbabwe revealed that most community leaders and healthcare professionals believed that healthcare personnel were the best individuals to disclose HIV status to children who are infected [56]. Similarly, a qualitative study conducted in Zimbabwe discovered that most adolescents preferred disclosure to take place in front of HCWs in a clinical setting since they have the professional abilities to handle any potential discomfort and have access to reliable information [57]. We therefore recommend that there be standardized rules in place that specify the relevant stakeholder to disclose HIV/AIDS to children living with HIV, based on the findings of the current study and the literature that has already been published. The OTLs further used multidisciplinary teams (MDT) as a source of support to the caregivers of HIV/AIDS to address various needs that require professional intervention. The multidisciplinary team refers to a group of different disciplines working together to improve the health and well-being of individuals [58].

The Elizabeth Glazier Paediatrics AIDS Foundation (EGPAF) guidelines indicate that if healthcare workers find caregivers who are reluctant to disclose their status to HIV/AIDS orphans, they must refer them to either social worker, nurses, psychologists, and other professionals for further counselling and support [59]. Within the context of this study, OTLs referred caregivers who had difficulty disclosing their HIV status of HIV/AIDS to psychologists for counselling and support. The quantitative study conducted by Nicastro et al. provided evidence in support of this finding, revealing that psychologists employed family group psychotherapy as a means of facilitating disclosure to families of children living with HIV [60]. The quality of life and overall well-being of orphaned children were found to be enhanced by group psychotherapy, which also decreased the children’s anxiety levels and boosted their resilience. Caregivers who were eligible to apply for social grants and did not have relevant documents were referred to social workers by OTLs for assistance. This finding agrees with the study conducted in SA, where social workers assisted orphans who did not have birth certificates to apply for social grants [61]. The author further reported that social workers also assisted orphans who did not have birth certificates to be admitted to schools. Consistent with previous studies, the study conducted in Zimbabwe reported that the Department of Social Welfare assisted caregivers in paying school fees and medical bills for HIV/AIDS orphans [59]. On the contrary, the qualitative study conducted in NWP, SA whereby caregivers of HIV/AIDS orphans were complaining about a lack of support from the social workers [62].

Furthermore, the authors claimed that one of the caregivers waited for the assistance of social workers to apply for a foster care grant for more than a year. Existing literature reported that the Department of Social Development in South Africa acknowledged that insufficient numbers of available social workers make it difficult to deliver social services where they are needed [63, 64]. The authors further revealed that social workers simply cannot deal with hundreds of thousands of foster care placements on top of the other services they need to provide [65, 66]. Based on the evidence of the current study and existing literature, we suggest that OTLs should collaborate with social workers to ensure the smooth running of the services that they render to caregivers.

HIV/AIDS orphans presenting with malnutrition and failure to thrive (FTT) were referred to dieticians for nutritional support. As defined by [67]. malnutrition is when people receive insufficient or excessive amounts of specific nutrients, which can negatively impact their health and result in an inability to thrive. According to [68] malnutrition is the most common reason children do not grow as well as they should. Similarly, the literature review demonstrated that ready-to-use therapeutic meals (RUTFs) improved the way severe acute malnutrition was treated in children living with HIV/AIDS by giving them access to foods that promote fast weight gain and are safe to use at home [69, 70].

In addition, a study conducted in Zimbabwe discovered that children with HIV who were given nutritional supplements by RUTFs for ten months, between the ages of twelve and thirty-six months, gained more weight of 51.2% than children who were not infected (26.0%) [71, 72]. The findings of this study support existing literature that children living with HIV who were malnourished and placed on nutritional supplements were showing huge progress. In this study, dieticians administered nutritional supplements for a certain period to bolster the weight of the orphans who were malnourished, and food parcels were provided for the entire family. This study described the support that should be given to caregivers if they discover instances of abuse and neglect involving HIV/AIDS orphans. The concepts “abuse” and “neglect” describe a scenario where a parent or caregiver causes physical, sexual, or psychological harm to children [73].

Suppose OTLs discover that an HIV/AIDS orphan has experienced abuse or neglect of any kind, whether it be psychological, emotional, physical, or sexual; in such cases, they should consult social workers to conduct an assessment and refer the case to law enforcement officers. Social workers’ responsibilities include conducting thorough assessments, offering initial counselling, drawing an intervention plan, and making therapy referrals [74]. In the study conducted in KwaZulu Natal province of South Africa social workers were not involved in the incident that involved an orphan who was sexually abused [75]. Despite the efforts made by the orphan child to report the incident to the caregiver, the sexual abuse incident continued. The orphan child decided to escalate the incident directly to the teacher at school who assisted her in reporting that to the law enforcement department. Consequently, the arrest of the perpetrator was affected by law enforcement officers however, this incident did not sit well with the caregiver who assaulted the orphan child for reporting the incident to the law enforcement officers [75].

The data reported here appear to support the assumption that orphaned children are sometimes not saved because caregivers who are supposed to offer them full protection are involved in the same acts of abuse against the orphaned children. A reasonable approach to tackle this issue could be to actively involve social workers as they are trained to handle such incidents. The effectiveness of the involvement of social workers in incidents of abuse of children has been exemplified in a study conducted in the Western Cape province of South Africa [64]. Children who were abused by the primary caregivers were removed from their care and placed in child and youth care centers (CYCC) [76]. Alternatively, the involvement of the social workers by OTLs could also aid in identifying people from the family who can foster the orphaned children who are found to be abused by the caregivers as reported by the current study. This action can assist in ensuring that the physical, psychological, and emotional well-being of the orphaned children are safeguarded.

Another interesting finding reported by this study was the use of adherence clubs and adolescent youth-friendly services by OTLs to improve the relationship between caregivers and HIV/AIDS orphans. This study highlighted that the Department of Health (DoH) established adherence clubs (ACs) throughout SA. Adherence clubs support cross-government initiatives to improve population health and well-being. The purpose of ACs is to accommodate the increasing number of stable patients undergoing treatment, guarantee the quality of care, and lessen the workload on healthcare personnel in the facilities [77]. The ACs are not explicitly used for HIV/AIDS but for other chronic conditions as well. Consistent with existing literature, a study conducted in Mpumalanga on community-based adherence clubs indicated they played a crucial role in reducing overcrowding in healthcare facilities, defaulter rates, improving treatment adherence, and reducing stigma and discrimination [78]. These results are similar to those reported in the study conducted in Haiti whereby the Kids Club program assisted caregivers and children living with HIV with psychosocial development and well-being through group interaction [79].

It has been reported that the Kids Club program was crucial in giving orphaned children the educational opportunities they needed to overcome their shyness, boost their self-esteem, give them encouragement and hope, and bring pleasure into their lives. Individuals who disclosed to the club that they were HIV-positive discussed how being a part of it had improved their knowledge of the virus, their ability to maintain their health, and their outlook on life [79]. Moreover, caregivers also reported how treatment treatment-taking behavior of orphaned children who attended the Kids Club improved. Similar results were reported in a recent study that the ACs smoothened the relationship between caregivers and HIV/AIDS orphans.

The OTLs further used adolescent youth-friendly services (AYFS) to educate orphaned adolescents about topics that affect their health and well-being. The findings of this study are aligned with previous studies that reported that peer support groups for adolescents and family clubs assisted adolescents in disclosing their status to significant others [80]. The author further reported that the initiative provided the caregivers of HIV/AIDS orphans with the opportunity to acquire more knowledge and skills on how to care for adolescents living with HIV, treatment adherence, and coping with stigma and discrimination. Similarly, a qualitative study conducted in five primary healthcare facilities in the SA revealed the effectiveness of a psychosocial support intervention (PSS) for adolescents on ART [81].

This initiative was implemented in an adolescent-friendly and safe space in the clinics in the form of social clubs. Interestingly, the authors stated that the PSS intervention promoted treatment adherence through peer support, health education, and relationships between adolescents, caregivers, and healthcare providers [81]. Another significant finding reported by the same authors was that the PSS intervention expedited full disclosure of HIV status to adolescents was also expedited by PSS intervention. Taken together, this finding supports the current study by highlighting the significance of support groups in reducing stigma and discrimination against caregivers and orphaned adolescents. Both studies further revealed the importance of support groups by harnessing the caring environment of the caregivers. In support of this finding, a qualitative study conducted in NWP of SA revealed that social clubs play a significant role in creating a supportive caring environment for caregivers of HIV/AIDS orphans by improving their psychological and emotional well-being [81]. To date, the success of AYFS has enhanced caregivers with multiple skills in how to care for HIV/AIDS orphans.

## Limitations

Recruitment of OTLs was challenging because the WBPHCOT program is championed by retired nurses due to the shortage of nurses in the NWP of SA. During data collection for this study, their contracts were expired therefore it was not easy to get hold of the OTLs. As a result of that, the planned completion date was not adhered to the researcher took time to collect data because the threshold for focus group was sub-minimal.

## Conclusions

This study provides new insights into the support provided by nurses, who works in a WBPHCOT as team leaders. The OTLs provide caregivers of children orphaned by HIV/AIDS in North West province with various dimensions of support. The support provided by outreach team leaders to the caregivers of children orphaned by HIV/AIDS play a critical role in ehancing their abilities to execute day to day care activities. Moreover, the support facilitated the rapport between caregivers and HIV/AIDS oprhans. The approach that was used by OTLs also curbed stigma and discrimination among both caregivers and HIV/AIDS orphans.

## Electronic supplementary material

Below is the link to the electronic supplementary material.

Supplementary Material 1

## Data Availability

Data collected in this study Data collected in this study is not available to the public. Only the researcher and study leaders have access to it. Moreover, the data are not publicly available due to privacy regulations. Currently, it is kept on a password-controlled computer to maintain confidentiality. Proper procedure should be followed if needed this include writing a formal letter to the corresponding author, to be submitted to NWU Health Research Ethics first for permission to be shared with other stakeholders.
