# Correction: Support provided by outreach team leaders to caregivers of HIV/AIDS orphans in the North-West province of South Africa

**DOI:** 10.1186/s12912-025-02933-0

**Published:** 2025-03-25

**Authors:** Boitumelo Joy Molato, Salaminah S. Moloko-Phiri, Magdalena P. Koen, Molekodi J. Matsipane

**Affiliations:** NuMIQ Research Focus Area, School of Nursing, Faculty of Health Sciences, North West University, Mahikeng Campus, Private Bag X2046 Mmabatho 2745, Mafikeng, South Africa

**Correction: BMC Nurs 23, 605 (2024)**

10.1186/s12912-024-02282-4

Following the publication of the original article [1], the author group identified an error in the first sentence of the Results section. The number of study participants was incorrectly listed as 37 when it should have been 27.

**Correct**

“A total of 27 professional nurses from five local municipalities voluntarily participated in this study”.

**Incorrect**

“A total of 37 professional nurses from five local municipalities voluntarily participated in this study”.

The original article [1] has been updated.
