# Supplementary material for: Support provided by outreach team leaders to caregivers of HIV/AIDS orphans in the North-West province of South Africa
Source: BMC Nurs. 2024 Aug 31;23:605. doi: 10.1186/s12912-024-02282-4 (PMC11366136; doi:10.1186/s12912-024-02282-4)
Supplement: Supplementary file 1 — Supplementary Material 1 [file 12912_2024_2282_MOESM1_ESM.pdf]

INTERVIEW SCHEDULE

ACTIVITY: SEMI-STRUCTURED FOCUS GROUP INTERVIEWS

POPULATION: OUTREACH TEAM LEADERS (PROFESSIONAL NURSES)

To obtain data, the following question will be asked:

- What is the support that you provide to caregivers of HIV/AIDS orphans in the North West province, South Africa?

Follow up questions using probing, clarifying and other communication techniques will be used to improve communication dynamics.
